# Sex-specific disruption of murine midbrain astrocytic and dopaminergic developmental trajectories following antenatal GC treatment

**DOI:** 10.1007/s00429-015-1049-0

**Published:** 2015-05-06

**Authors:** Simon McArthur, Ilse S. Pienaar, Sindhu M. Siddiqi, Glenda E. Gillies

**Affiliations:** Division of Brain Sciences, Imperial College London, Hammersmith Hospital Campus, Du Cane Road, London, W12 0NN UK; Department of Biomedical Sciences, Faculty of Science and Technology, University of Westminster, 115 New Cavendish Street, London, W1W 6UW UK; Department of Applied Sciences, Faculty of Health and Life Sciences, Northumbria University, Newcastle upon Tyne, UK

**Keywords:** Antenatal GC treatment, Midbrain, Astrocytes, Dopaminergic neurons, Neurobiological programming, Sex dimorphisms

## Abstract

The mammalian midbrain dopaminergic systems arising in the substantia nigra pars compacta (SNc) and ventral tegmental area (VTA) are critical for coping behaviours and are implicated in neuropsychiatric disorders where early life challenges comprise significant risk factors. Here, we aimed to advance our hypothesis that glucocorticoids (GCs), recognised key players in neurobiological programming, target development within these systems, with a novel focus on the astrocytic population. Mice received antenatal GC treatment (AGT) by including the synthetic GC, dexamethasone, in the mothers’ drinking water on gestational days 16–19; controls received normal drinking water. Analyses of regional shapes and volumes of the adult SNc and VTA demonstrated that AGT induced long-term, dose-dependent, structural changes that were accompanied by profound effects on astrocytes (doubling/tripling of numbers and/or density). Additionally, AGT induced long-term changes in the population size and distribution of SNc/VTA dopaminergic neurons, confirming and extending our previous observations made in rats. Furthermore, glial/neuronal structural remodelling was sexually dimorphic and depended on the AGT dose and sub-region of the SNc/VTA. Investigations within the neonatal brain revealed that these long-term organisational effects of AGT depend, at least in part, on targeting perinatal processes that determine astrocyte density and programmed cell death in dopaminergic neurons. Collectively, our characterisation of enduring, AGT-induced, sex-specific cytoarchitectural disturbances suggests novel mechanistic links for the strong association between early environmental challenge (inappropriate exposure to excess GCs) and vulnerability to developing aberrant behaviours in later life, with translational implications for dopamine-associated disorders (such as schizophrenia, ADHD, autism, depression), which typically show a sex bias.

**Electronic supplementary material:**

The online version of this article (doi:10.1007/s00429-015-1049-0) contains supplementary material, which is available to authorized users.

## Introduction

During late gestation, a natural rise in the bioavailability of endogenous glucocorticoid (GC) hormones produced by the foetal/maternal adrenal glands plays a fundamental role in the development of mammalian tissues and organs, including the brain (Fowden et al. 1998; Van den Bergh et al. 2005). This knowledge underpins the clinical practice of administering synthetic GCs to women at risk of pre-term delivery, to accelerate foetal lung maturation and infant survival (Liggins 1994). A single course of antenatal GC treatment (AGT) represents an unquestionable breakthrough in perinatal medicine, but repeated courses of AGT when pre-term delivery is delayed (approximately 50 % of cases) have been associated with significant effects on brain structure, neurological function and behaviour in childhood and adolescence (Talge et al. 2007; Finer et al. 2000; French et al. 2004; Baud and Sola 2007; Yeh et al. 2004; Asztalos 2012). However, the precise nature of GC-induced changes in the neural systems that regulate behaviour remains poorly characterised, but clearly has important implications from a clinical perspective.

In this regard, the midbrain dopaminergic systems have received particular interest. These pathways are critical regulators of normal adaptive behaviours, cognitive and sensorimotor functions (Williams and Goldman-Rakic 1995; Robbins and Everitt 1996; Bjorklund and Dunnett 2007), and several lines of evidence suggest that they are particularly vulnerable to in utero challenges, including exposure to inappropriate elevations in GC levels, due either to exogenous administration or maternal stressors (Pruessner et al. 2004; Weinstock 2008; Vuillermot et al. 2010; Gatzke-Kopp 2010; McArthur et al. 2007; Virdee et al. 2014). Furthermore, dysfunction of these pathways is seen in a range of neuropsychological disorders which have a putative neurodevelopmental component (Ben Amor et al. 2005; Szpir 2006; Khashan et al. 2008; Raikkonen et al. 2008; Bale et al. 2010; Charil et al. 2010). To date, the dopaminergic neurons themselves have been the main focus as potential targets of long-term GC programming in the brain. In contrast, effects on astrocytes, the main glial cell type of the brain, are unknown, despite our understanding that they play critical roles in normal brain development, structure, function and neuropathology (Barres 2008; Halassa and Haydon 2010; Schwarz and Bilbo 2012). Therefore, the present study sought to test our novel hypothesis that exposure AGT in late gestation will have enduring effects on the astrocytic populations of the adult rat substantia nigra pars compacta (SNc) and ventral tegmental area (VTA), which are, respectively, the origins of the nigrostriatal and mesocorticolimbic dopaminergic pathways. Furthermore, as substantial evidence shows that GC neurobiological programming leads to behavioural and endocrine effects which may be qualitatively and quantitatively different in males and females (Gabory et al. 2009; Luine et al. 2007; Lupien et al. 2009; Buss et al. 2007; Brummelte et al. 2012; Schwarz and Bilbo 2012; Sandman and Davis 2012; Reynolds 2013b; Kreider et al. 2005; Virdee et al. 2013, 2014), our experiments were conducted in both sexes.

Previously, we have shown in rats that AGT increased the cell counts and altered the topographical organisation of the dopaminergic neurons in the adult SNc and VTA (McArthur et al. 2005, 2006, 2007). During late gestation and the neonatal period, naturally occurring cell death via apoptotic mechanisms plays a key role in regulating adult numbers of dopaminergic neurons in the SNc (Jackson-Lewis et al. 2000; Oo et al. 2003; Burke 2004; Vitalis et al. 2005), but the factors regulating this process are unclear. Therefore, in the present study, we also tested our prediction (McArthur et al. 2007) that interference with developmental apoptosis in midbrain dopaminergic neurons represents a mechanism whereby AGT can permanently influence the adult population size. Mechanistically, it has been established that the effects of GCs in the immune and neuroendocrine systems are mediated via the signalling protein, annexin A1 (ANXA1) (Hannon et al. 2003; McArthur et al. 2010; John et al. 2008), but its role in the brain has received little attention (Solito et al. 2008). Therefore, the present study was performed in mice in order first, to establish whether our previous findings of AGT programming of the rat SNc/VTA dopaminergic neurons (McArthur et al. 2007, 2010) will be recapitulated mice, and then to use genetically manipulated mice to investigate whether a potential mechanism for the effects of AGT on the midbrain dopaminergic neurons involves ANXA1.

## Materials and methods

### Animals

All procedures were performed under a licence issued by the United Kingdom Animals Home Office. CBa/ca mice were used for the majority of experiments, whilst annexin A1 (ANXA1) null mice (Hannon et al. 2003) and C57BL/6 wild-type mice were also used, where specified. Mice were bred in-house and housed in the Imperial College facility, Hammersmith campus. The animals were maintained under controlled temperature (21–23 °C), humidity (63 %) and lighting (lights on 08.00–20.00 h), with free access to standard mouse chow and drinking water. Mice were mated overnight and matings were confirmed by the presence of vaginal plugs the following morning. The day following overnight mating was defined as embryonic day (E) 0. Pregnancy was confirmed approximately 10 days later via palpation. Pregnant mice were housed 5 per cage until gestational day (GD) 15, after which they were caged singly. The day of birth was defined as postnatal day 0 (P0). Offspring were weaned at P21, after which time the sexes were housed separately (5 animals per cage) and allowed to grow to adulthood, during which time they received only standard husbandry and no further treatment.

### Dexamethasone treatment regimes

In accordance with previously published protocols (McArthur et al. 2005, 2006, 2007), a non-invasive method for late gestational administration of AGT was employed. The method, comprising of adding dexamethasone sodium phosphate (Faulding Pharmaceuticals Plc., Royal Leamington Spa, UK) to the drinking water of the pregnant mice, overcomes the potential confounding effects of stress associated with handling the animals and injection of drugs. The pregnant mice receiving AGT were divided into two groups, with one receiving a lower dose of dexamethasone (AGT_L_, 0.5 µg/ml in the drinking water) from GDs 16–19, and the other receiving the higher dose (AGT_H_, 1.0 µg/ml in the drinking water) for the same period of time. Dams in the control group received normal drinking water. There were no group differences in the volumes of water consumed, which were 15.54 ± 0.45, 12.00 ± 1.46 and 13.09 ± 1.5 ml (mean ± standard error of mean, SEM) for controls, AGT_L_, and AGT_H_, respectively, representing a daily dose of approximately 150–175 µg/kg (AGT_L_) or 300–350 µg/kg (AGT_H_). As discussed previously (McArthur et al. 2006, 2007), the levels of endogenous GCs that are required for normal lung maturation during late gestation are in the range of physiological ‘stress’ levels and, from a pharmacokinetic standpoint, the optimal dose of dexamethasone for mimicking this in rats has been estimated to be 288 µg/kg/day over GD 18–20 (Samtani et al. 2006a, b). Although equivalent pharmacokinetic data are not available for the mouse, the chosen dose of AGT_L_ and AGT_H_ span a relevant range and, moreover, are in the range used in clinical perinatal medicine (Jobe and Soll 2004; Yeh et al. 2004). This treatment had no significant effect on maternal behaviour or the offspring’s adult body weight (McArthur et al. 2007). When analysing the effects of AGT on the adult SNc and VTA, the potential effects of litter of origin were minimised using one male/female per litter per treatment group (*n* = 6 per group).

### Tissue collection and processing

#### Adult brains

At P67 ± 2, mice underwent terminal anaesthesia via intraperitoneal injection of 100 µl sodium pentobarbitone. This was followed by transcardial perfusion using 0.9 % heparinized saline (until the fluid exiting the cut right atrium was entirely clear), followed by 20–30 ml of 4 % paraformaldehyde dissolved in 0.1 M phosphate-buffered saline (PBS, 0.1 M NaH_2_–PO_4_·2H_2_O, 0.1 M Na_2_HPO_4_·12H_2_O, 0.15 M NaCl, all reagents purchased from VWR International, Poole, UK). Brains were rapidly removed from the skull and post-fixed in 4 % paraformaldehyde (PFA) for ~23 h, then cryoprotected by immersion in 30 % sucrose for ~72 h, before freezing them on dry ice and storing them at −80 °C until sectioning. A 1 in 4 series of coronal sections (20 µm) were cut using a cryostat (−22 °C, Bright Instruments Ltd., Huntingdon, UK) and the free-floating sections stored (−20 °C) in antifreeze solution (0.1 M NaH_2_PO_4_·H_2_O, 0.05 M Na_2_HPO_4_, 0.15 Mm NaCl, 50 % v/v ethanediol, 1 % w/v polyvinylpyrrolidone, 0.1 % w/v NaN_3_, VWR International).

#### P2 brains

To investigate for developmental apoptosis in dopaminergic neurons, a separate set of coronal mouse brain sections were collected from mice euthanised at P2, with tissue preparation adapted for the immature brains. Briefly, mice were decapitated, brains removed, fixed in 4 % PFA for 4 days, cryoprotected in 30 % sucrose for 2 days, frozen on dry ice and stored (−80 °C). Cryostat sections (20 µm) were cut directly onto poly-d-lysine coated glass slides (−22 °C) and stored at −80 °C.

### Double-antigen immunofluorescence

#### Adult brains

Double-antigen immunostaining for the dopaminergic neuronal marker, tyrosine hydroxylase (TH) and the astrocyte marker, glutamine synthetase (GS) (Vardimon et al. 1999; Halassa and Haydon 2010) was performed on free-floating sections. In brief, after rinsing sections with PBS, they were incubated for 1 h at room temperature (RT) in 10 % normal goat serum (NGS, Serotec, Oxford, UK) to saturate non-specific binding sites. Sections were then permeabilized with 0.05 % Triton X-100 (VWR International, UK) in 1 % NGS in PBS for 5 min, followed by incubation overnight on a shaker at 4 °C with a combination of monoclonal mouse anti-mouse TH (1:4000, Millipore, UK) and polyclonal rabbit anti-mouse GS (1:5000, Abcam, Cambridge, UK). The following morning, sections were washed in PBS for 3 × 5 min, before incubation for 1 h at RT in the dark with a mixture of Alexa Fluor 594 dye (red) goat anti-mouse IgG for identifying TH-immunoreactive (IR) neurons, and Alexa Fluor 488 dye (green) goat anti-rabbit IgG (H + L) both at dilution factor 1:500, Invitrogen, Paisley, UK) for visualising GS-IR cells. Finally, a 0.5 % solution of Sudan Black B (Sigma, Poole, UK) in 70 % ethanol was applied to the sections for 5 min. Since Sudan Black B binds lipofuscin present in neurons, the treatment reduces autofluorescence (Romijn et al. 1999). Sections were then rinsed and allowed to air dry on gelatin-coated slides before being mounted with Vectorset mounting medium (Vector Laboratories, UK) in a rostrocaudal direction.

#### P2 brains

Coronal sections were subjected to immunofluorescent detection of TH (as described above) followed by nuclear counterstaining with a 0.03 % solution of fluorescent 4′,6-diamidino-2-phenylindole (DAPI, Vector Laboratories) in 0.01 M PBS for 20 min prior to mounting. These sections were used for visualisation of chromatin condensation and nuclear chromatin clumps, which are characteristic of apoptosis (Oo and Burke 1997; Oo et al. 2003). Apoptotic cell death in dopaminergic neurons was further confirmed using double-antigen immunofluorescence for TH in addition to detection of activated caspase-3 using a polyclonal rabbit antibody (1:1000; Cell Signaling Technology Inc., USA). The secondary antibody used for detection of activated caspase-3 was Alexa Fluor 488-conjugated goat anti-rabbit IgG (H + L) (Invitrogen, Paisley, UK; both 1:500), with the sections left to incubate for 1 h at RT. The sections were washed well in PBS, and then processed for nuclear counterstaining with DAPI, as described above. All sections were allowed to air dry on gelatin-coated slides, and then mounted with Vectorset mounting medium (Vector Laboratories, UK) in a rostrocaudal direction.

### Regional volumes, cell counts and neuronal size in adult brains

**Fig. 1:**
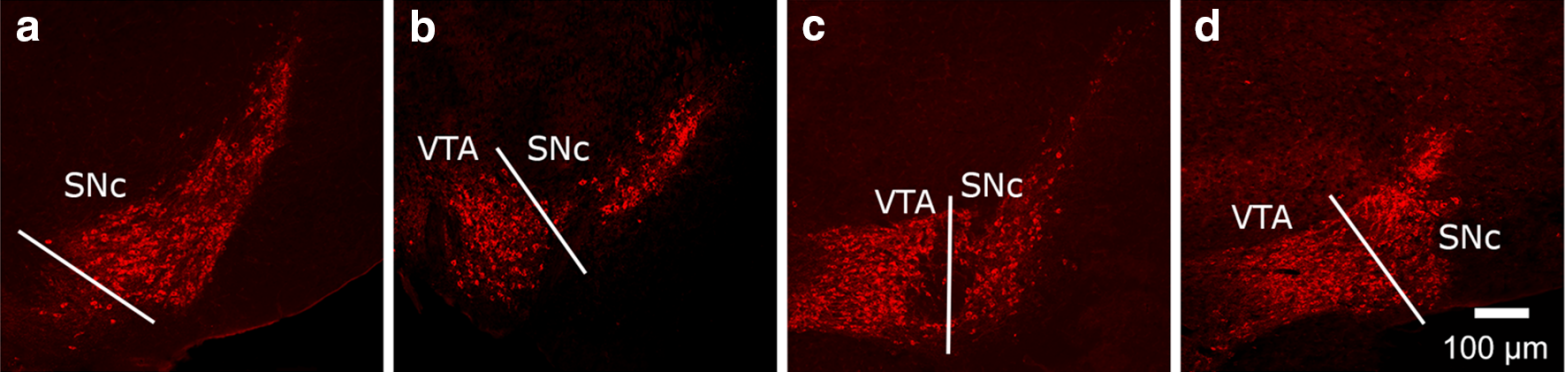
Representative images, moving in a rostrocaudal direction, at the different levels of the *SNc* (**a**–**d**) and *VTA* (**b**–**d**)

Immunoreactive cells were visualised at 15× magnification using a Nikon TE2000U epifluorescence microscope fitted with red and green band-pass filters, a Plan Achromat 10× objective lens, and a 1.5× magnifying lens, linked to a Hamamatsu C4742-95 CCD camera (Hamamatsu Photonics UK, Welwyn Garden City, UK) and an Apple Macintosh G5 computer, running Openlab 5.5 software (Perkin-Elmer, Coventry, UK). Images were coded and stored in a way such that the person performing the cell analysis was unaware of the treatment groups. Each section was projected onto a computer monitor and the contours of the SNc and VTA were delineated by the TH-IR neuronal groups and by referring to visual anatomical landmarks and to the mouse brain atlas of Franklin and Paxinos (Franklin and Paxinos 2008). The SNc can be clearly distinguished from the surrounding non-DA regions of the thalamus dorsally, and the substantia nigra pars reticulata (SNr) ventrally by the presence of TH-IR cells, and from the adjacent VTA by the third cranial nerve tract that runs between the two nuclei. The VTA was delineated by the TH immunopositive region bordered medially by the interfascicular nucleus and the interpeduncular nucleus, and dorsally by the parabrachial-pigmented nucleus. Where the SNr was analysed as a control region, it was defined as the region of tissue ventral to the SNc and dorsal to the glia limitans on the ventral surface of the brain. To detect any regional differences in volume/shape and cell distribution through the SNc/VTA, sections containing the nuclei were divided into four levels (A–D), each spanning 200–250 µm, in a manner analogous to that which we have used previously for analysing the rat SNc/VTA (McArthur et al. 2007), beginning at the anatomical level represented at around −2.7 mm relative to bregma, where the SNc TH-IR cells first appear. Representative images at each level are shown in Fig. 1. Level A was defined solely as the SNc, due to difficulties in distinguishing the very rostral parts of the VTA, which begin to mix with the SNc at this level; level D was defined as the most caudal level for analysis to avoid the problem of distinguishing the dopaminergic populations of the retrorubral field, which mix with the caudal SNc (Fu et al. 2012). Using this delineation, the majority of the TH-IR cells bodies, which are used to define the contours of the nuclei within which the astrocytes (GS-IR cells) are counted, are included in the 4 levels of our analysis, and the TH-IR at levels more caudal to level D is largely due to cell fibres, rather than perikarya, that slowly coalesce to form the MFB.

Regional volumes were calculated using Cavalieri’s method (McArthur et al. 2007) where the cross-sectional area for each level (A–D) of the SNc/VTA, defined by the presence of TH-IR cells, was measured, multiplied by 20 (the section thickness before staining of sections cut at 20 µm) and then quadrupled (to account for staining every 4th section); the overall volume was the sum of the volumes for levels A–D (SNc) and B–D (VTA). The process of fixing, staining and mounting results in a degree of tissue shrinkage, but as this value is constant across all treatment groups, it was not accounted for in the analysis (McArthur et al. 2007).

To estimate the total cell population per level per animal, IR cells within the whole region of interest were counted manually using the count function plug-in available in ImageJ (Rasband, US National Institutes of Health, version 1.4., http://rsb.info.nih.gov/ij/). This two-dimensional counting technique is most appropriate for relatively small nuclei such as the SNc and VTA because it allows for the inclusion of all cells across the whole of the coronal plane, providing a large sampling window for accurate assessment (McArthur et al. 2007; Virdee et al. 2014). It has also been demonstrated that such empirically derived counts of dopaminergic neurons in the SNc, using serial reconstruction, are not significantly different from those obtained using stereological methods (Baquet et al. 2009).

To estimate TH-IR cell size, six such cells per level per animal were randomly chosen from digital images of coded sections, selecting only cells in which the nucleus could be readily identified. From such images, the cross-sectional area was calculated (McArthur et al. 2007). The mean cell areas per level per animal were pooled to form the group means (*n* = 6).

### Morphological analysis of immature brains

A z-stack of optical sections was captured from each mouse brain tissue slice that had been counterstained with DAPI, using a Leica TCS SP5 inverted confocal laser scanning imaging system (Leica Microsystems, Heidelberg, Germany) equipped with a Leica DFC 320 digital camera, and powered by a Chameleon Ultra-II MP laser (Coherent Inc., CA, USA) and visualised with red–green–blue RGB lasers (Leica Microsystems). The confocal z-stacks were taken in 0.78 μm steps with an apochromat 63×/1.4 NA oil-immersion objective lens, in sequential scanning mode. All parameters (pinhole, contrast, gain and offset) were held constant for all sections from the same experiment. The captured images were saved with Northern Eclipse software (Empix Imaging Inc., Mississauga, Canada), exported as TIFF files, and processed post-capture using Adobe Photoshop software (version 7.0), adjusting them for levels of brightness and contrast. Images were merged automatically using the built-in functions available in Photoshop software.

We found that the accuracy of delineating the SNc and VTA at P2 was relatively poor, so the region was measured as a single entity. Apoptotic profiles were quantified by scanning the entire SNc/VTA for each section. TH-IR neurons were classified as apoptotic when their DAPI-stained nuclei appeared condensed, round, brighter and/or fragmented, compared to healthy nuclei (Ahmadi et al. 2003). Moreover, apoptotic nuclei were identified by detection of nuclear chromogen condensation, a morphological alteration indicative of apoptosis (Oo and Burke 1997; Blomgren et al. 2007). Other studies have confirmed that apoptotic profiles assessed using similar methods represent unbiased counts appropriate for the quantification of natural cell death in dopaminergic neurons (Oo and Burke 1997; Oo et al. 2003; Jackson-Lewis et al. 2000). Images were analysed post-capture using ImageJ software. Nuclear profiles surrounded by TH-IR cytoplasm were counted in 5 tissue sections per animal, and the proportion of cells exhibiting chromatin condensation was calculated. Group means were calculated from these values. Confirmation of apoptotic cell death was made by co-localisation of activated caspase-3 IR within TH-IR cells.

### Statistical analysis

Statistical analyses were carried out using SigmaPlot scientific data analysis and graphing software package (v. 12.5, Systat Software Inc, London, UK). Preliminary analysis assessed whether data were normally distributed, by performing a Kolmogorov–Smirnov test. Global analysis of variances (ANOVAs) were initially performed on data groupings for each parameter measured, these being cell number and nucleus volume with sex, AGT regimen and level of the SNc/VTA comprising as factors. Data were then subdivided according to the interacting factors, and lower order effects of sex and AGT treatment were examined by means of a one-way ANOVA test, followed by post hoc analysis by means of a Tukey’s honestly significant difference (HSD) test. A probability value of *p* < 0.05 was considered significant.

## Results

Global three-way ANOVA revealed significant three-way interactions between sex, level and treatment for the parameters described below, as presented in Online Resource 1.

### AGT effects on regional volumes in the adult SNc and VTA

**Fig. 2:**
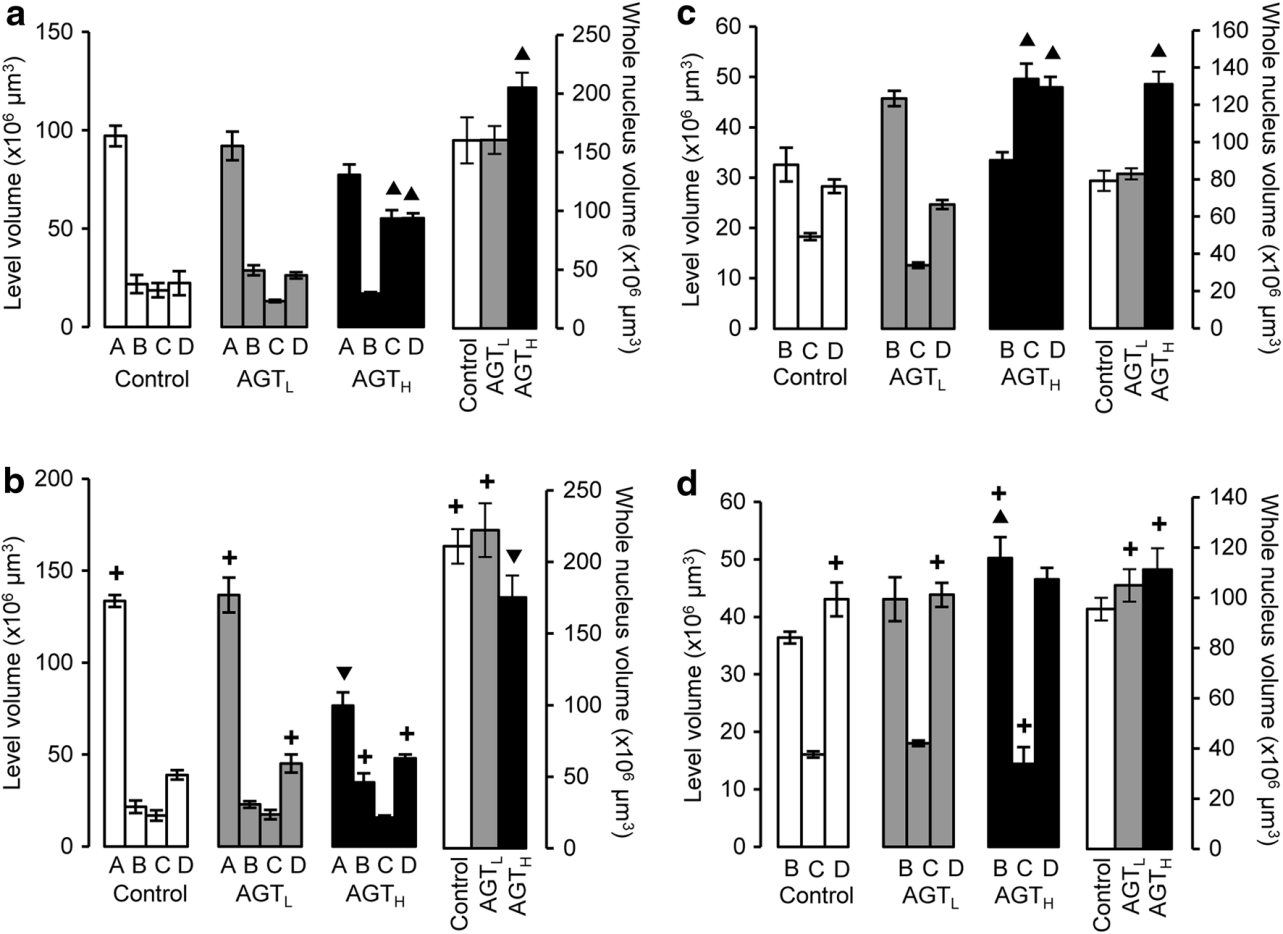
The volume of individual levels A–D and total volume for the SNc (**a**, **b**) and VTA (**c**, **d**) of male (**a**, **c**) and female (**b**, **d**) mice in adulthood following antenatal treatment with dexamethasone via the maternal drinking water on embryonic days 16–19 (*grey bars* lower dose antenatal GC treatment, AGT_L_, 0.5 µg/ml; *black bars* higher dose antenatal GC treatment, AGT_H_, 1.0 µg/ml) compared to the control offspring of dams receiving normal drinking water (*open bars*). Data are mean ± SEM, *n* = 6 animals per treatment group. *Up filled triangle*, *down filled triangle* dexamethasone treatment had a significant effect, *p* < 0.05, to increase or decrease volume, respectively, compared with control animals; *plus* a significant sex difference, *p* < 0.05 vs. males in the same treatment group

The overall shape of the SNc was similar in control male and female mice, but the sum of the volumes at each level was significantly greater in females compared with males by 31 %, with the major difference occurring at level A (Fig. 2a, b, open bars). AGT_L_ had no effect in either sex (Fig. 2a, b, grey bars). In males, AGT_H_ induced a marked overall change in the shape of the nucleus, as seen by an increase in volumes at levels C and D, resulting in a net increase (28 %) in overall volume (Fig. 2a, black bars). In contrast, in females, AGT_H_ decreased overall SNc volume by 17 % due to a substantial effect only at level A (Fig. 1b, black bars).

The overall VTA volume was similar in control male and female mice although a significantly greater volume at level D in females indicates a sex difference in the overall shape (Fig. 2c, d open bars). As for the SNc, AGT_L_ had no effect in either sex, whereas AGT_H_ increased total volume in males by 64 % due to significant effects at C and D, resulting in a marked change in the overall shape of the nucleus (Fig. 2c, black bars). However, in females there was no overall effect of AGT_H_ (Fig. 2d, black bars).

### AGT effects on astrocytes in the adult SNc and VTA

**Fig. 3:**
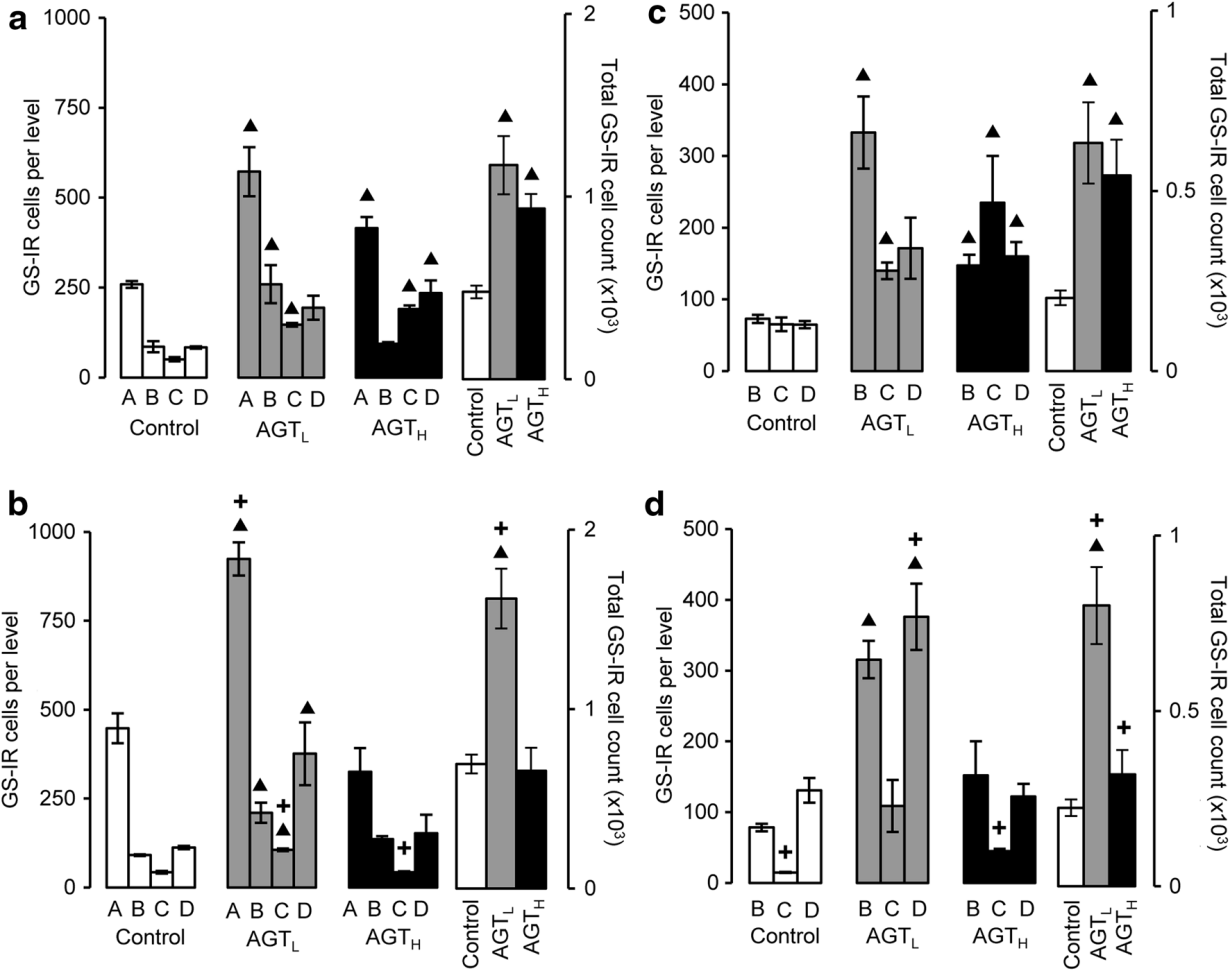
GS-IR cell counts at the individual levels A–D and total cell counts over the entire SNc (**a**, **b**) and VTA (**c**, **d**) of male (**a**, **c**) and female (**b**, **d**) mice in adulthood following antenatal treatment with dexamethasone via the maternal drinking water on embryonic days 16–19 (*grey bars* lower dose antenatal GC treatment, AGT_L_, 0.5 µg/ml; *black bars* higher dose antenatal GC treatment, AGT_H_, 1.0 µg/ml) compared to the control offspring of dams receiving normal drinking water (*open bars*). Data are mean ± SEM, *n* = 6 animals per treatment group. *Up filled triangle*, *down filled triangle* dexamethasone treatment had a significant effect, *p* < 0.05, to increase or decrease cell counts, respectively, compared with control animals; *plus* a significant sex difference, *p* < 0.05 vs. males in the same treatment group

The GS-IR cell count and distribution within the SNc were similar in control males and females (Fig. 3a, b, open bars). In both sexes, AGT_L_ resulted in a two- to threefold increase in the estimated total GS-IR cell count, as well as the counts at individual levels (Fig. 3a, b, grey bars). Females were affected to a greater extent than males, leading to a significant sex difference. This effect was sustained in males after AGT_H_, but in females AGT_H_ had no significant effect (Fig. 3a, b, black bars).

The estimated total GS-IR cell counts made within the VTA were also similar in control males and females, although a notably lower count at level B in females would indicate a significant sex difference in the distribution of astrocytes throughout the VTA (Fig. 3c, d, open bars). In both sexes, AGT_L_ induced a remarkable three- to fourfold increase in the estimated total adult GS-IR cell numbers, due to a particularly marked effect at level B in males and at both levels B and D in females (Fig. 3c, d, grey bars). There was thus a sex difference in regional responsiveness of the astrocytes, which led to the estimated total GS-IR cell count in the adult female VTA being double that seen in the male after exposure to AGT_L_. AGT_H_ also greatly increased in the estimated total GS-IR cell numbers in males, and, in contrast to AGT_L_, significant effects were seen at all 3 levels, indicating dose effects on regional responsiveness. In females, however, AGT_H_ was without effect. This sexually dimorphic pattern of responses in the VTA mirrored that seen in the SNc.

To evaluate the relationship between regional volumes (Fig. 2) and astrocyte numbers (Fig. 3), GS-IR cell density is presented in Table 1. This reveals that exposure to AGT_L_ caused a two- to threefold increase in astrocyte density at all levels of the SNc and VTA in both sexes, except for the VTA at level C in females, where an almost sevenfold increase led to a significant sex difference. In contrast, AGT_H_ failed to alter GS-IR cell density in any region or sex, except for the female VTA at level C, where the increase in density (threefold) was approximately half that seen with the lower dose of AGT. Notably, neither AGT_L_ nor AGT_H_ affected adult GS-IR cell density in the neighbouring SNr in either sex (Table 1), indicating that the effect in the SNc and VTA was not a uniform, non-specific effect throughout the brain.

**Table 1:** Influence of AGT on GS-IR cell density in the adult brain

	GS-IR cell density (×10^4^ cells/mm^3^)			
	Level	Control	AGT_L_	AGT_H_
**SNc**				
Male	A	0.27 ± 0.01	0.65 ± 0.08*	0.55 ± 0.07
	B	0.42 ± 0.09	1.06 ± 0.31*	0.55 ± 0.02
	C	0.28 ± 0.04	1.10 ± 0.05*	0.38 ± 0.09
	D	0.39 ± 0.05	0.78 ± 0.17*	0.44 ± 0.07
Female	A	0.34 ± 0.04	0.69 ± 0.07*	0.44 ± 0.10
	B	0.54 ± 0.11	0.95 ± 0.15*	0.38 ± 0.09
	C	0.26 ± 0.02	0.62 ± 0.16*	0.27 ± 0.03
	D	0.28 ± 0.02	0.97 ± 0.25*	0.33 ± 0.13
**VTA**				
Male	B	0.23 ± 0.02	0.72 ± 0.10*	0.44 ± 0.03
	C	0.36 ± 0.07	1.12 ± 0.11*	0.51 ± 0.09
	D	0.23 ± 0.02	0.69 ± 0.11*	0.32 ± 0.03
Female	B	0.22 ± 0.02	0.76 ± 0.10*	0.24 ± 0.04
	C	0.09 ± 0.01^+^	0.61 ± 0.23*^,+^	0.31 ± 0.06^+^
	D	0.28 ± 0.06	0.85 ± 0.11*	0.27 ± 0.04
SNr				
Male	–	1.66 ± 0.23	2.01 ± 0.33	1.84 ± 0.12
Female	–	2.08 ± 0.19	2.20 ± 0.20	1.75 ± 0.37

Density of GS-IR cells throughout the SNc, VTA and SNr of male and female mice in adulthood following treatment with dexamethasone via the maternal drinking water prenatally on embryonic days 16–19 (AGT_L_ 0.5 µg/ml; AGT_H_ 1.0 µg/ml) compared to the control offspring of dams receiving normal drinking water. Data are mean ± SEM, *n* = 6 animals per treatment group

* Significant effect of treatment, *p* < 0.05 for dexamethasone-treated vs. control animals

^+^Significant sex difference, *p* < 0.05 vs. males in the same treatment group

### AGT effects on TH-IR cells in the adult SNc and VTA

**Fig. 4:**
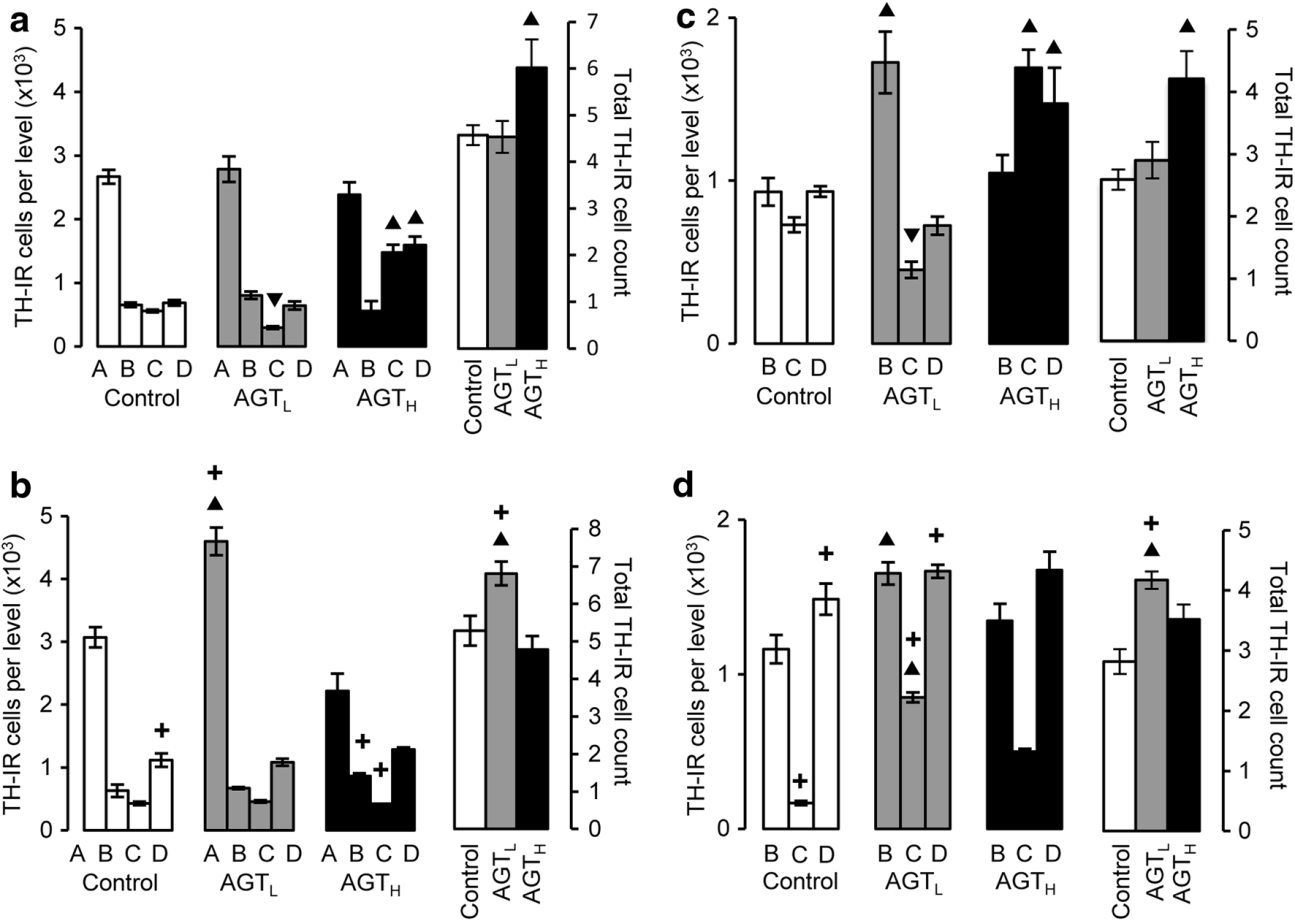
TH-IR cell counts at the individual levels A–D and total cell counts over the entire SNc (**a**, **b**) and VTA (**c**, **d**) of male (**a**, **c**) and female (**b**, **d**) mice in adulthood following antenatal treatment with dexamethasone via the maternal drinking water on embryonic days 16–19 (*grey bars* lower dose antenatal GC treatment, AGT_L_, 0.5 µg/ml; *black bars* higher dose antenatal GC treatment, AGT_H_, 1.0 µg/ml) compared to the control offspring of dams receiving normal drinking water (*open bars*). Data are mean ± SEM, *n* = 6 animals per treatment group. *Up filled triangle*, *down filled triangle* dexamethasone treatment had a significant effect, *p* < 0.05, to increase or decrease cell counts, respectively, compared with control animals; *plus* a significant sex difference, *p* < 0.05 vs. males in the same treatment group

TH-IR cell counts within the adult SNc were similar in control males and females (Fig. 4a, b open bars). AGT_L_ had no overall effect in male mice, but significantly increased the estimated total cell count in females due to a predominant effect at level A. In contrast, AGT_H_ increased counts at C and D in male mice, leading to an overall significant increase (32 %) in the estimated total TH-IR cell count, whereas females were unaffected by AGT_H_.

In the VTA, TH-IR cell counts were similar in control males and females, but at the individual levels C and D counts were significantly lower or higher, respectively, in females compared with males, indicating a sex difference in the distribution of dopaminergic neurons throughout the nucleus (Fig. 4c, d). In males AGT_L_ significantly increased counts at level B and decreased them at C, with no net overall effect, whereas AGT_H_ increased estimated total counts by 62 % due to effects at C and D. Females, in contrast, responded only to AGT_L_ with significantly increased counts at B and C.

Table 2 shows that, with only one exception, neither dose of AGT affected regional TH-IR cell density in the adult SNc and VTA of either sex. The one exception was the female VTA at level C, where TH-IR cell density was increased approximately fourfold or threefold after AGT_L_ or AGT_H_, respectively. There are thus sex effects on regional responsiveness.

**Table 2:** Influence of AGT on TH-IR cell density in the adult brain

	TH-IR cell density (×10^4^ cells/mm^3^)			
	Level	Control	AGT_L_	AGT_H_
**SNc**				
Male	A	2.76 ± 0.12	3.07 ± 0.2	3.11 ± 0.27
	B	3.36 ± 0.87	2.86 ± 0.23	3.23 ± 0.74
	C	3.19 ± 0.53	2.23 ± 0.13	2.77 ± 0.47
	D	3.27 ± 0.7	2.46 ± 0.22	2.87 ± 0.16
Female	A	2.71 ± 0.32	3.42 ± 0.26	2.87 ± 0.09
	B	3.47 ± 1.21	3.00 ± 0.25	2.55 ± 0.26
	C	2.60 ± 0.27	2.72 ± 0.32	2.70 ± 0.18
	D	2.93 ± 0.39	2.49 ± 0.24	2.69 ± 0.12
**VTA**				
Male	B	2.92 ± 0.41	3.75 ± 0.45	3.15 ± 0.40
	C	3.98 ± 0.12	3.61 ± 0.36	3.46 ± 0.33
	D	3.31 ± 0.27	2.97 ± 0.30	3.09 ± 0.52
Female	B	3.20 ± 0.28	3.97 ± 0.43	2.72 ± 0.35
	C	1.05 ± 0.12^+^	4.72 ± 0.09*	3.58 ± 0.59*
	D	3.50 ± 0.32	3.83 ± 0.17	3.60 ± 0.21

Density of TH-IR cells at each level throughout the substantia nigra pars compacta (SNc) and ventral tegmental area (VTA) of male and female mice in adulthood following treatment with dexamethasone via the maternal drinking water prenatally on embryonic days 16–19 (AGT_L_ 0.5 µg/ml; AGT_H_ 1.0 µg/ml) compared to the control offspring of dams receiving normal drinking water. Data are mean ± SEM, *n* = 6 animals per treatment group

* Significant effect of treatment, *p* < 0.05 for dexamethasone-treated vs. control animals

^+^Significant sex difference, *p* < 0.05 vs. males in the same treatment group

Measurement of cross-sectional areas of TH-IR cell bodies established that there were regional or sex differences, with the average respective values (mean ± s.e.m, µm^2^) for males and females being 113.5 ± 2.6 and 112.1 ± 2.3 for the SNc and 125.3 ± 7.3 and 114 ± 2.9 for the VTA. No significant interactions of treatment groups were seen. Thus, regional changes in volume or cell density cannot be attributable to any effects of AGT on TH-IR cell sizes.

### AGT influences at P2

**Fig. 5:**
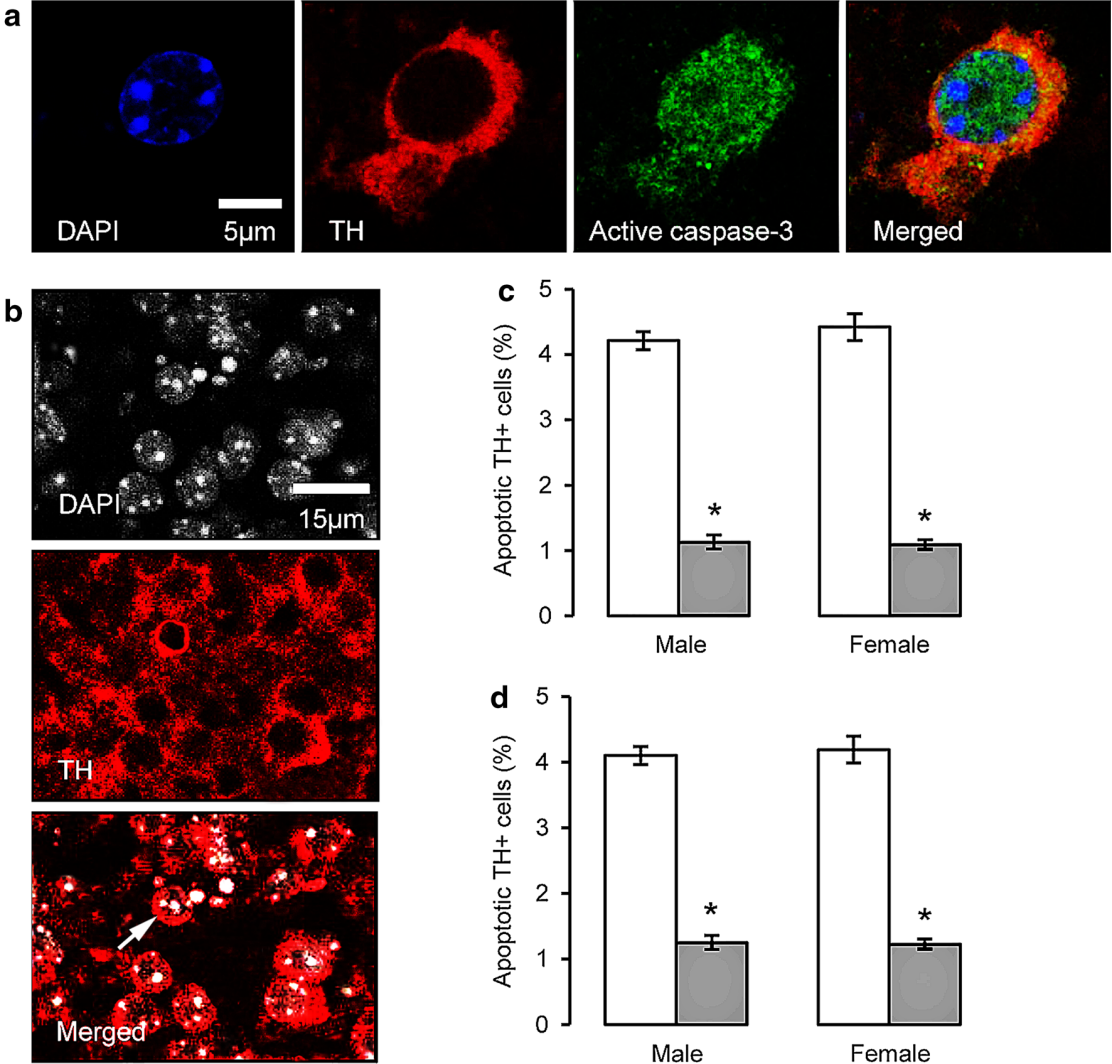
A proportion of murine TH-IR cells undergo apoptotic changes on postnatal day P2, as identified by immunostaining for activated caspase-3 (**a**), or by the presence of nuclear condensation (**b)**, *white arrow* an example of pronounced nuclear condensation. TH-IR cell apoptosis was quantified in the combined SNc and VTA at postnatal day P2 in male and female wild-type (**c**) or annexin A1 null (**d**) mice treated with dexamethasone via the maternal drinking water antenatally on embryonic days 16–19 (*grey bars* 0.5 µg/ml), and compared to the control offspring of dams receiving normal drinking water (*open bars*). Data are mean ± SEM, *n* = 6 animals per treatment group. *Asterisk* a significant effect of treatment, *p* < 0.05 for dexamethasone-treated vs. control animals

**Fig. 6:**
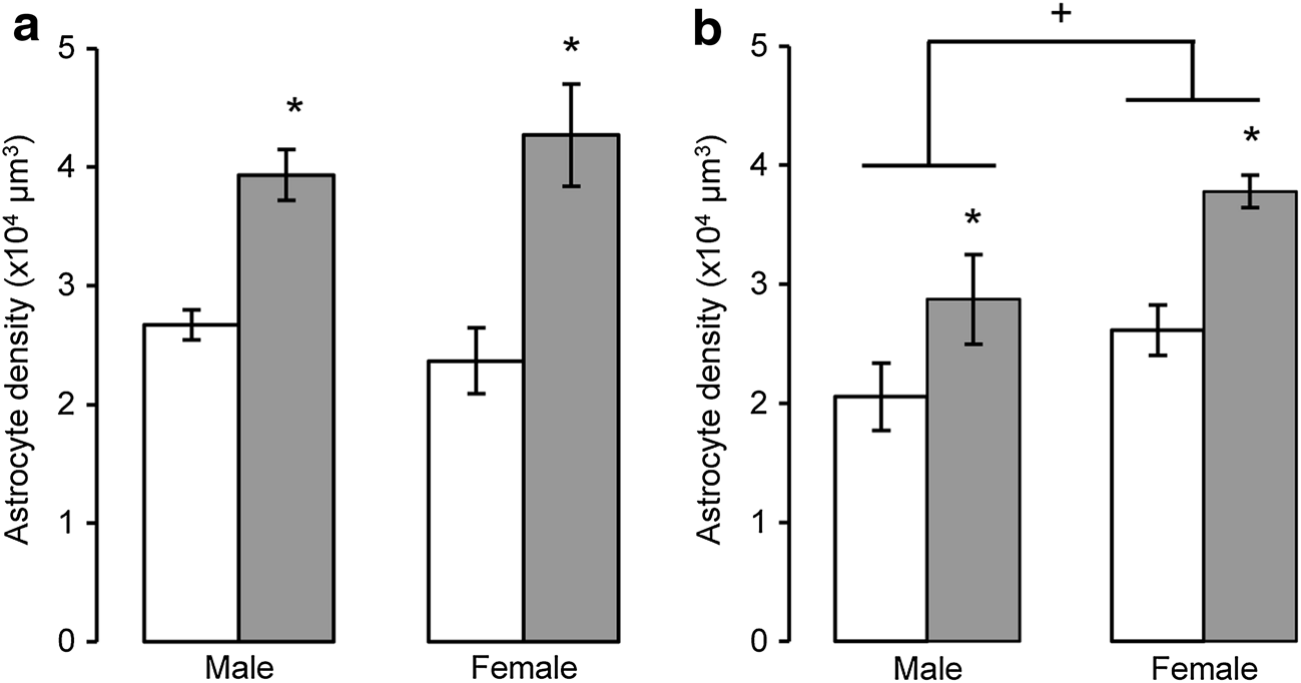
Density of GS-IR cells in the combined SNc and VTA (**a**) and adjacent SNr (**b**) of male and female mice at postnatal day P2 following treatment with dexamethasone via the maternal drinking water antenatally on embryonic days 16–19 (*grey bars* 0.5 µg/ml) compared to the control offspring of dams receiving normal drinking water (*open bars*). Data are mean ± SEM, *n* = 6 animals per treatment group. *Asterisk* a significant effect of treatment, *p* < 0.05 for dexamethasone-treated vs. control animals. *Plus* a sex difference, *p* < 0.05

As a peak of programmed cell death (PCD) via apoptosis has been reported at P2 (Burke 2004), the effects of AGT_L_ on apoptotic markers in TH-IR were assessed at this time point. Figure 5a provides an example of chromogen condensation (blue DAPI fluorescence) in the nucleus of a TH-IR neuron (red fluorescence), which is also co-localised with active caspase-3 (green fluorescence), as confirmed by the merged image. At a lower magnification, Fig. 5b illustrates that the cells exhibiting nuclear chromatin condensation are also positive for TH-IR, and all analyses indicated that apoptotic markers were restricted to TH-IR cells, with no evidence of apoptosis in glial cells. In both wild-type and ANXA1-null mice, AGT_L_ markedly suppressed apoptosis in the male and female SNc/VTA, indicating no role for ANXA1 in programmed cell death of the midbrain dopaminergic neurons (Fig. 5c, d). At P2, the density of GS-IR cells was significantly increased in both sexes by AGT_L_ in the SNc/VTA region (Fig. 6a), in the SNr (Fig. 6b) and also in the red nucleus (data not shown).

A qualitative summary of the main effects of the two doses of AGT on the estimated numbers and density of astrocytes and dopaminergic neurons in the SNc and VTA of adult male and female rats, as well as neonatal apoptosis in these regions is provided in Table 3.

**Table 3:** Summary of principal findings

	SNc				VTA			
	Males		Females		Males		Females	
	AGT_L_	AGT_H_	AGT_L_	AGT_H_	AGT_L_	AGT_H_	AGT_L_	AGT_H_
Volume	=	↑ C, D	=	↓ A	=	↑ C, D	=	=
GS-IR cell counts	↑↑ A–C	↑↑ A, C, D	↑↑ A–D	=	↑↑ B, C	↑↑ B, C, D	↑↑ B, D	=
GS-IR cell density	↑↑ A–D	↑ A	↑↑ A–D	↑ A	↑↑ A–D	↑ B	↑↑ A–D	↑ C
TH-IR cell counts	=	↑ C, D	↑ A	=	=	↑ C, D	↑ B, C	=
TH-IR cell density	=	=	=	=	=	=	=	↑↑ C
Apoptosis	↓	NA	↓	NA	↓	NA	↓	NA

The qualitative changes in the adult mouse SNc and VTA consequent on antenatal treatment with dexamethasone via the maternal drinking water on embryonic days 16–19 (lower dose antenatal glucocorticoid treatment, AGT_L_, 0.5 µg/ml; higher dose antenatal glucocorticoid treatment, AGT_H_, 1.0 µg/ml) are compared to the control offspring of dams receiving normal drinking water. = denotes no change with treatment; ↑ a significant increase compared with relative control; ↑↑ a significant increase by >100 % compared with relative control; ↓ a significant decrease compared with relative control; NA this parameter was not assessed in this treatment group. A, B, C or D indicates the level(s) where a significant effect was seen

## Discussion

Although GCs are widely accepted as potent mediators of neurobiological programming in the brain (Seckl and Holmes 2007; Reynolds 2013a), little is known of their impact on astrocytic populations. The present study has identified dose-dependent effects of AGT on the structure and volume of the mouse SNc and VTA that are associated with marked effects on the number, density and distribution of astrocytes, as well as neurons, in these regions. Moreover, these effects were sexually dimorphic, depending on the dose of AGT as well as the sub-region of the SNc/VTA, and were super-imposed on intrinsic sex differences in volume, shape and/or distribution of astrocytes and dopaminergic neurons in control animals. These findings thus provide new data highlighting astrocytes as AGT targets that contribute to the structural remodelling of the SNc and VTA as a result of inappropriate exposure to GCs in late gestation. They also add new data in support of the mounting evidence that the impact of early life challenges within the brain is sexually dimorphic (Gabory et al. 2009; Luine et al. 2007; Lupien et al. 2009; Buss et al. 2007; Brummelte et al. 2012; Schwarz and Bilbo 2012; Sandman and Davis 2012; Reynolds 2013b; Kreider et al. 2005; Virdee et al. 2013, 2014). Additionally, the data demonstrate for the first time that AGT-dependent programming of dopaminergic cell numbers occurs in mice and follows a similar pattern to that which we have reported previously in rats (McArthur et al. 2007; Virdee et al. 2014). This comparison identifies AGT-induced neurobiological programming with the midbrain dopaminergic systems as a robust, interspecies phenomenon, thereby laying the basis for future work in transgenic animals to better understand the mechanisms underlying environmental/epigenetic programming of key pathways controlling adaptive behaviours and their likely susceptibility to malfunction.

### Regional volumes and shape in the adult SNc/VTA

The present study revealed an intrinsic sex difference in the overall volume (SNc) and shape (SNc and VTA) of the midbrain nuclei in control animals. As sex differences in regional brain volumes strongly associate with behavioural differences in humans and rodents (Cahill 2006; Cosgrove et al. 2007; Swaab 2008; Forger 2009), these findings provide structural evidence in support of functional sex differences (behavioural, neurochemical and neurodegenerative responses) that have been reported for these regions in the normal brain (Gillies et al. 2014a, b; Becker and Hu 2008). The effects of AGT on regional volume appeared to be dose-dependent, with only the group exposed to the higher dose showing neuroanatomical disturbances. Notably, however, the effect in males was to increase the overall SNc and VTA volumes (effects at caudal levels C, D), whereas in females overall volume fell in the SNc (changes rostrally at level A), with no effects in the VTA, thereby exaggerating the sex dimorphisms seen in control animals. To date, structural changes induced by early life GC exposure have been reported for the hippocampus, amygdala, prefrontal cortex and hypothalamus, with evidence for different effects in males and females as well as for links to neuropsychiatric disorders (Uno et al. 1994; Vythilingam et al. 2002; Gilbertson et al. 2002; Sapolsky 2002; Coe et al. 2003; Geuze et al. 2005; Buss et al. 2007, 2012; Lupien et al. 2009; Rao et al. 2010; McEwen and Gianaros 2010; Charil et al. 2010; Garcia-Caceres et al. 2010). The present study therefore highlights the potential for SNc/VTA volume changes to be used as a sensitive, non-invasive biomarker for disorders where early life environmental challenges, as well as dopaminergic malfunction, contribute to their aetiologies.

### Astrocytes

#### Adult SNc/VTA

To our knowledge, this is the first study to demonstrate that brief prenatal exposure to GCs has dramatic, enduring consequences for the number and density of astrocytes in the adult brain. In both sexes, AGT_L_ increased SNc/VTA astrocyte counts. However, contrary to expectations, this did not affect the volume of these regions. This demonstrates that cellular changes can occur without overt structural changes and highlight the importance of coupling functional/biochemical markers with volumetric measurements (discussed above) as potential aids to diagnosis or the understanding of the aetiology of brain disease (Pruessner et al. 2004). Astrocyte counts were similarly increased in the male group treated with the higher dose of AGT, but, unlike the lower dose, regional volumes also expanded such that astrocyte density was largely unaffected. Whether astrocyte size accounts for these differences remains to be determined. Factors that regulate astrocyte size are largely unknown, but this is critical to their function as each occupies a large, unique spatial domain, associating with several hundred dendrites and 100,000 synapses in the rodent brain, and 20-fold more synapses in the human brain (Freeman 2010). Although GS-IR is very effective for visualising the immediate astrocyte cell body for cell counting purposes, it provides little information on astrocyte size. The alternative use of glial fibrillary acidic protein (GFAP), a cytoskeletal protein, as an astrocytic marker would offer no further advantages because it reveals approximately only 15 % of total astrocytic volume (Freeman 2010; Halassa and Haydon 2010). Further studies are therefore needed to discover the precise contributions of astrocytes to AGT-induced structural changes.

In contrast to findings in males, astrocytes of the female SNc/VTA were largely unresponsive to AGT_H_. Together, these sexually dimorphic AGT-induced alterations in the astrocytic environment, which would inevitably impact on midbrain dopaminergic function, suggest a novel mechanism which may underpin the sex-specific effects of early environmental disturbances on adult behaviours. In support of this, astrocyte disruptions are increasingly being recognised in DA-dependent CNS pathologies, which exhibit sex differences as well as a developmental component, including schizophrenia, depression and neurodegenerative disorders (Barres 2008; Halassa and Haydon 2010; Rappold and Tieu 2010).

#### Neonatal SNc/VTA

The effect of AGT_L_ to increase GS-IR cell density was manifested by P2. As most astrocytes are produced postnatally (Bandeira et al. 2009), and cessation of AGT at E19 ensures that the mice are dexamethasone-free by P21, these results establish that postnatal astrocyte development is highly sensitive to prenatal events, the course of which can be altered by AGT. In the developing vertebrate CNS neural precursor cells first generate neurons and then undergo a “neurogenic-to-gliogenic switch” late in gestation in rodents (Freeman 2010), but the triggers of this switch are unknown. However, the GC receptor (GR) is widely expressed in astrocytes, GCs can up-regulate GS expression, and maturation of the adrenal gland has been linked to glial differentiation, at least in the chick retina (Vardimon et al. 1999; Zschocke et al. 2005). Our results in mice therefore add to and extend the evidence that GCs are important for astrocyte ontogeny and suggest the intriguing hypothesis that they have a role in regulating neural precursor cell fate.

AGT_L_ influences on astrocyte density in the SNc/VTA were qualitatively similar in both sexes at P2 and in adulthood, but quantitatively values fell five- to tenfold between these periods. A contrasting pattern was seen in the SNr, where both inherent sex differences and a positive effect of AGT on astrocyte density at P2 were absent in adulthood, and developmental changes in density were relatively small (~30 %). The mechanisms underlying these changes require further investigation, but the current data suggest that important processes in midbrain development intervene between the neonatal period and adulthood, and are region- and sex-specific. Together, our novel findings in the adult and neonatal SNc/VTA clearly highlight the need for a better understanding of neuronal–glial interactions in the midbrain dopaminergic systems, as well astrocyte vulnerability to hormonal disruption, which remain relatively under-investigated.

### Dopaminergic neurons

#### Adult SNc/VTA

The present work demonstrated that AGT permanently increased TH-IR neuron numbers in the mouse SNc and VTA in both sexes, although sex dimorphisms in regional responsiveness and dose effects occurred, resulting in notable sex differences in the number and distribution of dopaminergic neurons after AGT. These results recapitulate our previous findings in the rat of an AGT-induced increase in the population size and topographical re-organisation of the midbrain dopaminergic neurons (McArthur et al. 2005, 2007), with implications for their connectivity and function (Brodski et al. 2003; Bjorklund and Dunnett 2007; Roeper 2013). We have also demonstrated that other neurobiological indicators of dopaminergic transmission, such as striatal levels of the DA transporter, the DA Type 1 receptor and amphetamine-stimulated DA release, also changed markedly in animals exposed to AGT, but in a diametrically opposite direction in males and females, which we have proposed represent sexually dimorphic adaptive mechanisms (Virdee et al. 2014). Given the importance of astrocytes for normal functioning of the SNc and VTA systems, the present data suggest that astrocytic programming by in utero GC exposure may contribute to these sexually dimorphic compensatory processes which underpin behavioural resistance, susceptibility or instability [allostatic load (McEwen 1998; Beauchaine et al. 2011)].

It remains to be determined whether the effects of AGT on GS-IR cells could be related to, or compensate for, effects on TH-IR cells. The comparative data summary in Table 3 illustrates that the regional effects of AGT on astrocyte numbers in the adult brain do not match concomitant changes in dopaminergic neuron numbers and there is no concordance between the directional change in astrocytic/neuronal numbers and volume in males and females. The data thus indicate a complex, sex-specific pattern where the influences of AGT on dopaminergic neurons and astrocytes may well be independent.

#### Neonatal SNc/VTA

Neurogenesis, specification and migration of the midbrain dopaminergic neurons are largely complete in the midbrain before commencement of AGT (Smidt and Burbach 2007). Hence, the present study indicates that GCs must influence already committed neurons during late gestation. Furthermore, our data on apoptosis within the dopaminergic neurons at P2 suggest that, mechanistically, this involves downstream suppression of the wave of ‘classical’ neuronal PCD that occurs just after birth (Burke 2004). As several important actions of GCs are mediated via ANXA1 (John et al. 2008; Hannon et al. 2003), and ANXA1 has a putative role in apoptosis by promoting non-inflammatory phagocytosis of cell debris derived from various cell types (McKanna 1995; Yona et al. 2006; McArthur et al. 2010), we reasoned that ANXA1 could be involved in the mechanism by which GCs modulate natural cell death in the developing brain. However, the ability of AGT to suppress expression of apoptotic makers in wild-type and ANXA1 null mice to a similar extent argues against ANXA1-dependent mechanisms in this process, which therefore requires further investigation.

Although AGT_L_ suppressed apoptosis in the SNc/VTA to a similar extent in both sexes at P2, this dose increased the adult TH-IR count only in females. The female data therefore support the premise that PCD in early life regulates DA neuron numbers in the adult SNc (Burke 2004). Longitudinal studies will be required to investigate why a similar effect did not endure into adulthood in males, but we propose that intervening developmental processes differentially affected by AGT in males and females have additional influences on the final neuron numbers. In support of this, amygdala nuclei volumes and numbers of glia and neurons may be increased or decreased by in utero programming depending on whether they were assessed before P25 or after P45, highlighting that outcomes may differ according to the time of testing (Kraszpulski et al. 2006). The nature of the intervening processes remains speculative, but emerging evidence implicates adolescence as a critical window, in addition to the classical neonatal period, when elevations in gonadal steroid hormones can organise the brain and programme adult behaviours in humans and animals (Sisk and Zehr 2005; McArthur et al. 2011; Gillies and McArthur 2010; McCarthy et al. 2008; Juraska et al. 2013). Together, our findings highlight a need to understand the impact of sex hormones and their interactions with stress/stress hormones on the ontogeny not only of the midDA neurons, but also their associated astrocytes.

### Implications and conclusions

As the multi-drug resistance protein, *P*-glycoprotein, extrudes dexamethasone from the adult brain, access of peripherally administered dexamethasone to the brain has been questioned. However, this protein has not been detected in the rodent brain before P7 (Matsuoka et al. 1999). Additionally, GRs are expressed in the basal ganglia, including astrocytes, from E15 in rodents, and GCs can up-regulate GS expression during development (Vardimon et al. 1999; Zschocke et al. 2005; Diaz et al. 1998). Together, these observations support the view that AGT may directly target the cells of the SNc and VTA during development.

In providing a direct link between GC actions in the developing brain and cytoarchitectural disturbances in the adult brain, the current study suggests novel mechanisms which may underpin the strong association between early environmental challenges and vulnerability to neuropsychopathologies, such as schizophrenia, attention deficit/hyperactivity disorder, autism spectrum disorders, substance abuse, Parkinson’s disease, anxiety and depression (Ben Amor et al. 2005; Szpir 2006; Khashan et al. 2008; Raikkonen et al. 2008; Thomas et al. 2009; Hu et al. 2004; Barlow et al. 2007), which involve the ascending DA systems and typically show a sex bias (Aleman et al. 2003; Becker and Hu 2008; Biederman et al. 2002; Baron-Cohen et al. 2005; Gillies et al. 2014a, b). In support of this, we have recently demonstrated that specific behaviours that have psychopathological relevance and are known to involve midbrain dopaminergic systems in numerous species, including rats, mice and humans, are differentially affected by AGT in male and female rats (Virdee et al. 2014). These relate to female behaviours (motivation, arousal), which are altered in depression [more prevalent in women (Kessler 2003)] and male behaviours (pre-attentional processing/pre-pulse inhibition of the startle response), which are affected in male, but not female, schizophrenic subjects (Kumari et al. 2008). Given our evidence for the similarity in AGT programming of the SNc/VTA dopaminergic neurons between mice and rats, it would seem reasonable to suggest that cytoarchitectural re-programming would lead to functional consequences in mice, just as it did in rats. Nonetheless, future studies will be required to ascertain this, and to consider the alternative hypothesis that astrocyte plasticity may represent compensatory mechanisms to preserve function, which is a characteristic of the midbrain dopaminergic systems (Golden et al. 2013; Virdee et al. 2014).

Although precise information on the differential pharmacokinetics between humans, rats and mice is lacking, certain considerations, discussed in detail elsewhere (McArthur et al. 2006, 2007), support the view that the doses of dexamethasone selected in the current study hold physiological and translational relevance. Specifically, they were in the low clinical range used in perinatal medicine (Ballard and Ballard 1995; Jobe and Soll 2004) and also fall within the range required for inducing normal lung maturation in the rat (Samtani et al. 2006a, b). Furthermore, treatment was administered at a stage in mouse brain development which approximates to the latter part of the second trimester of human gestation (Clancy et al. 2001), when AGT is administered in cases of threatened premature birth (Liggins 1994). Our findings therefore highlight the sensitivity of the SNc/VTA neural systems to AGT and underscore the concerns for incurring neurological deficits after repeated courses of AGT, which remain in practice, despite recommendations to the contrary and the lack of evidence for any proven clinical benefits (Baud and Sola 2007; Seckl and Holmes 2007; Murphy et al. 2008). As GC programming mechanisms are thought to be similar in humans and experimental species (Seckl and Holmes 2007), the data also support the validity of our model for predicting potential neuropathological changes after early inappropriate exposure to GCs, and for testing strategies to reverse such effects.

## Electronic supplementary material

Below is the link to the electronic supplementary material.

Supplementary material 1 (PDF 267 kb)
