# Supplementary material for: Sex-specific disruption of murine midbrain astrocytic and dopaminergic developmental trajectories following antenatal GC treatment
Source: Brain Struct Funct. 2015 May 6;221:2459–75. doi: 10.1007/s00429-015-1049-0 (PMC4884206; doi:10.1007/s00429-015-1049-0)
Supplement: Supplementary file 1 — Supplementary material 1 (PDF 267 kb) [file 429_2015_1049_MOESM1_ESM.pdf]

Brain Structure and Function

Electronic Supplementary Material

Sex-specific disruption of murine midbrain astrocytic and dopaminergic developmental trajectories following antenatal GC treatment

Simon McArthur[1,2,3] , Ilse S. Pienaar[1,2], Sindhu M. Siddiqi[1], Glenda E. Gillies[1*]

1. Division of Experimental Medicine, Department of Medicine, Imperial College London, Hammersmith Hospital Campus, Du Cane Road, London W12 0NN
2. Joint first authorship
3. Current address: William Harvey Research Institute, Barts and The London School of Medicine and Dentistry, Charterhouse Square, London EC1 6BQ, UK

Address all correspondence and requests for reprints to:

* Professor Glenda E. Gillies, Division of Experimental Medicine, Department of Medicine, Imperial College London, Hammersmith Campus, Du Cane Road, London W12 0NN; E-mail: g.gillies@imperial.ac.uk; Telephone: (44)-020-7594 7050.

Supplemental Table 1: Results of global 3-way ANOVA for various parameters in the SNc

	Parameter measured	Source of variation	F	p
SNc	Nucleus volume	Sex	$F_{1,80}$=14.39	<0.001
		Level	$F_{3,80}$=388.28	<0.001
		Treatment	$F_{2,80}$=0.38	0.69
		Sex x Level	$F_{3,80}$=14.79	<0.001
		Sex x Treatment	$F_{2,80}$=9.79	0.002
		Treatment x Level	$F_{6,80}$=17.11	<0.001
		Sex x Level x Treatment	$F_{6,80}$=5.85	<0.001
	GS-IR cell number	Sex	$F_{1,80}$=2.71	0.106
		Level	$F_{3,80}$=103.51	<0.001
		Treatment	$F_{2,80}$=43.78	<0.001
		Sex x Level	$F_{3,80}$=6.14	0.001
		Sex x Treatment	$F_{2,80}$=7.25	0.002
		Treatment x Level	$F_{6,80}$=8.42	<0.001
		Sex x Level x Treatment	$F_{6,80}$=3.72	0.004
	TH-IR cell number	Sex	$F_{1,80}$=7.19	0.009
		Level	$F_{3,80}$=304.00	<0.001
		Treatment	$F_{2,80}$=1.39	0.257
		Sex x Level	$F_{3,80}$=13.42	<0.001
		Sex x Treatment	$F_{2,80}$=13.54	<0.001
		Treatment x Level	$F_{6,80}$=14.32	<0.001
		Sex x Level x Treatment	$F_{6,80}$=4.83	<0.001
	TH-IR cell density	Sex	$F_{1,80}$=0.24	0.628
		Level	$F_{3,80}$=0.92	0.439
		Treatment	$F_{2,80}$=0.71	0.494
		Sex x Level	$F_{3,80}$=0.06	0.980
		Sex x Treatment	$F_{2,80}$=1.03	0.362
		Treatment x Level	$F_{6,80}$=0.92	0.483
		Sex x Level x Treatment	$F_{6,80}$=0.21	0.972
	GS-IR cell density	Sex	$F_{1,80}$=1.27	0.264
		Level	$F_{3,80}$=1.77	0.165
		Treatment	$F_{2,80}$=30.81	<0.001
		Sex x Level	$F_{3,80}$=0.53	0.665
		Sex x Treatment	$F_{2,80}$=0.50	0.611
		Treatment x Level	$F_{6,80}$=0.93	0.482
		Sex x Level x Treatment	$F_{6,80}$=0.74	0.622

Global three-way ANOVAs were performed for the substantia nigra pars compacta (SNc) to investigate the effects of antenatal glucocorticoid treatment compared with controls on the counts of cells immunoreactive for tyrosine hydroxylase (TH-IR) and glutamine synthetase (GS-IR), nucleus volumes, TH-IR and GS-IR cell distribution, and the TH-IR and GS-IR cell density.

Supplemental Table 2: Results of global 3-way ANOVA for various parameters in the VTA

	Parameter measured	Source of variation	F	p
**VTA**	Nucleus volume	Sex	$F_{1,60}$=2.96	0.092
		Level	$F_{2,60}$=97.27	<0.001
		Treatment	$F_{2,60}$=31.25	<0.001
		Sex x Level	$F_{2,60}$=27.97	<0.001
		Sex x Treatment	$F_{2,60}$=13.39	<0.001
		Treatment x Level	$F_{4,60}$=9.63	<0.001
		Sex x Level x Treatment	$F_{4,60}$=20.20	<0.001
	GS-IR cell number	Sex	$F_{1,60}$=0.12	0.726
		Level	$F_{2,60}$=11.84	<0.001
		Treatment	$F_{2,60}$=51.18	<0.001
		Sex x Level	$F_{2,60}$=10.27	<0.001
		Sex x Treatment	$F_{2,60}$=5.80	0.006
		Treatment x Level	$F_{4,60}$=6.07	0.001
		Sex x Level x Treatment	$F_{4,60}$=2.78	0.041
	TH-IR cell number	Sex	$F_{1,60}$=2.33	0.134
		Level	$F_{2,60}$=41.62	<0.001
		Treatment	$F_{2,60}$=13.85	<0.001
		Sex x Level	$F_{2,60}$=23.93	<0.001
		Sex x Treatment	$F_{2,60}$=11.60	<0.001
		Treatment x Level	$F_{4,60}$=11.53	<0.001
		Sex x Level x Treatment	$F_{4,60}$=9.10	<0.001
	TH-IR cell density	Sex	$F_{1,60}$=0.005	0.945
		Level	$F_{2,60}$=0.151	0.860
		Treatment	$F_{2,60}$=8.48	0.001
		Sex x Level	$F_{2,60}$=3.06	0.057
		Sex x Treatment	$F_{2,60}$=6.78	0.003
		Treatment x Level	$F_{4,60}$=3.08	0.025
		Sex x Level x Treatment	$F_{4,60}$=4.86	0.002
	GS-IR cell density	Sex	$F_{1,60}$=6.46	0.015
		Level	$F_{2,60}$=0.94	0.401
		Treatment	$F_{2,60}$=72.70	<0.001
		Sex x Level	$F_{2,60}$=6.92	0.003
		Sex x Treatment	$F_{2,60}$=0.22	0.803
		Treatment x Level	$F_{4,60}$=0.48	0.753
		Sex x Level x Treatment	$F_{4,60}$=1.42	0.246

Global three-way ANOVAs were performed for the ventral tegmental area (VTA) to investigate the effects of antenatal glucocorticoid treatment compared with controls on the counts of cells immunoreactive for tyrosine hydroxylase (TH-IR) and glutamine synthetase (GS-H), nucleus volumes, TH-IR and GS-IR cell distribution, and the TH-IR and GS-IR cell density.
